# Integrated Single-Cell and Spatial Profiling of MMP Gene Expression in Colorectal Cancer

**DOI:** 10.64898/2026.04.17.719089

**Published:** 2026-04-22

**Authors:** Nicholas A. Danese, Shan Kurkcu, Marina Bleiler, Klea Nito, Alan Kuo, Daniel W. Rosenberg, Masako Nakanishi, Charles Giardina

**Affiliations:** 1 Department of Molecular and Cell Biology, University of Connecticut, Storrs, CT 06269-3125; 2 Center for Molecular Medicine, University of Connecticut Health, Farmington, CT 06030-3101

## Abstract

Increased matrix metalloproteinase (MMP) expression has long been recognized as a common feature of colorectal cancers (CRCs), yet less is known about how these enzymes interact to impact cancer progression. Taking advantage of single-cell and spatial transcriptomic data, we analyzed the cell-type-specific and spatial expression of MMPs in CRCs. Distinct colon cancer-associated fibroblast (CAF) subtypes were found to express different MMP combinations, including MMP1/3-expressing and MMP11-expressing CAFs. Conversely, myeloid cells (monocytes, macrophages, and dendritic cells) expressed varying levels of the “myeloid MMPs” 9, 12, and 14, which correlated closely with secretory gene expression. Finally, a small population of cancer cells expressed high levels of MMP7. The MMP7-expressing cancer cells frequently co-expressed MMP1, MMP14, and several Wnt-related genes, consistent with a cancer cell type at high risk of malignancy and metastasis. Spatial transcriptomic data showed MMP expression in discernible clusters driven in part by cell-type localization, including fibroblast-heavy stromal regions and inflammatory cell hubs. Epithelial-rich areas showed subregions of MMP7-expressing cancer cells, including areas where cancer cell and myeloid MMP expression overlap. Tumors showed a wide variation in MMP1-expressing CAFs, a variation reflected in primary CAF cell lines. In vitro, MMP1 expression was a stable phenotype that persisted through multiple rounds of division. MMP1-expressing CAFs were frequently positioned at the stromal interface, suggesting a role in facilitating cell movement across the tumor boundary. Our analysis indicates that cell-type and positional MMP expression varies between tumors and may play a role in determining lesion progression and cancer spread.

## Introduction

Colorectal cancer (CRC) is the third most commonly diagnosed malignancy worldwide and a leading cause of cancer-related mortality, with an estimated 1.9 million new cases and over 900,000 deaths annually [1]. While the overall incidence of CRC is lowering, it is increasing in younger populations, particularly in high-income countries, emphasizing the need for improved understanding of CRC etiology and pathophysiology [1, 2]. CRCs consist of both genetically transformed epithelial cells and a diverse stromal compartment comprised of fibroblasts, endothelial cells, and inflammatory cell infiltrates. Cancer cells exhibit uncontrolled proliferation and invasive capacity, while stromal cells serve as key modulators of tumor progression in part through their roles in angiogenesis, immune and inflammatory responses [3]. The reciprocal interactions between cancer cells and stromal components actively shape the tumor microenvironment (TME), which can significantly impact cancer growth and progression.

Matrix metalloproteinases (MMPs) are a family of zinc-dependent endopeptidases that mediate extracellular matrix degradation and thus play a pivotal role in tissue remodeling under both physiological and pathological conditions. MMPs cleave ECM components, which in turn can impact cell attachment, signaling, and movement into, out of, and through a tissue. MMPs can therefore affect myriad events in cancer, ranging from metastasis to immune surveillance. These enzymes are classified based on their substrate specificity and structural characteristics into collagenases (MMP1, MMP8, MMP13, MMP18), gelatinases (MMP2, MMP9), stromelysins (MMP3, MMP10, MMP11, MMP12), matrilysins (MMP7, MMP26), and membrane-type MMPs (MT-MMPs, e.g., MMP14, MMP15, MMP16, MMP25)[4, 5]. Each group cleaves a selection of ECM components, with some overlap between groups. In addition, MMPs can cleave non-ECM proteins to modulate their activity, including cytokines and membrane receptors that play a central role in CRC promotion. MMP expression increases in inflamed and neoplastic tissues, with much of this regulation occurring at the transcriptional level. MMPs are initially expressed as zymogens (pro-MMPs) that require cleavage for activation, often by other MMPs, and are inhibited by tissue inhibitors of metalloproteinases (TIMPs), providing two additional layers of regulation. MMP cleavage by other MMPs establishes activation cascades, wherein one MMP activates another. For example, MMP3 can activate both MMP7 and MMP9, while MMP2 is activated by MMP14 [6–8]. Matrilysins, particularly MMP7, play a key role in CRC by degrading basement membrane components and activating MMP2 and MMP9, reinforcing an auto-regulatory proteolytic network that sustains tumor progression [6]. This cooperative enzymatic interplay between MMPs enhances ECM degradation to effect cellular trafficking and metastasis. Understanding the interplay between various MMP subtypes within CRC tissue is therefore important for understanding numerous events of CRC progression.

While MMPs have been extensively studied, their spatial expression within CRC (and other cancers) remain less well defined. Advances in single-cell RNA sequencing and spatial transcriptomics now enable high-resolution mapping of MMPs in CRC, providing insights into their localization and potential functional interactions. This knowledge could lead to a better understanding of how these enzymes collectively influence ECM composition and cancer progression. This information could also be leveraged to develop novel therapeutic approaches that exploit cancer-associated changes in MMP expression, either through inhibitors that halt lesion growth or as targets for cancer-seeking therapies and prodrugs.

## Results

### MMP expression in CRC

**Figure 1:**
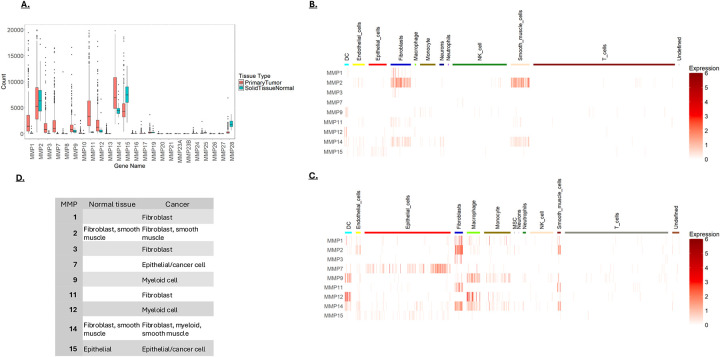
**A)** MMP expression in colorectal tissue. Data was retrieved from TCGA and analyzed to identify the most highly expressed MMPs in noncancerous mucosa and primary CRC lesions. Shown are the normalized counts from RNA-seq data. **B&C)** Single cell RNA-seq (scRNA-seq) data was used to determine MMP expression patterns in cell types isolated from normal colon tissue **(B)** and cancerous colon tissue **(C)**. Single cell RNA sequencing was obtained from the NCBI GEO database (accession code GSE132465). Normalized expression levels of MMPs are shown in rows with cell type indicated by the color bar across the top of the heatmap. **D)** Comparative cellular expression of MMPs in normal and tumor microenvironments. This table summarizes the results presented in **1B** and **1C**.

TCGA data was analyzed to identify the MMPs highly expressed in CRC. This analysis revealed high expression of MMPs 1, 2, 3, 7,9,11,12,14 and 15 (Fig 1A). Most of these MMPs are expressed at higher levels in cancer than normal tissue, although MMPs 2, 4, and 15 are expressed at relatively high levels in normal tissue. Since CRCs are populated by numerous cell types that can impact lesion progression, we determined the MMP expression profiles in different cell types within healthy and cancer tissue using scRNA-seq data. In normal tissue, scRNA-seq data showed fibroblasts (FBs) and smooth muscle cells (SMCs) to be the highest MMP expressors, with MMP2 being the highest expressed isoform. In CRC, many more cell types express MMPs; there are notable increases in epithelial cells and myeloid cells, in addition to increased expression in FBs and SMCs. A table summarizing cell-type MMP expression in normal and CRC tissue is shown.

### MMP expression in CAFs and SMCs

**Figure 2:**
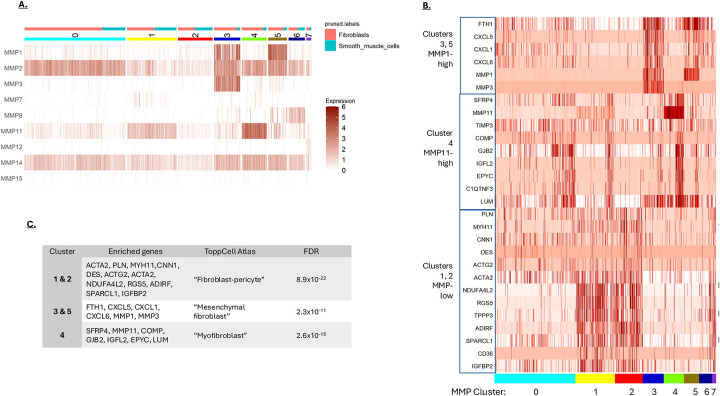
**A)** MMP-based clustering of mesenchymal cells from CRC. Fibroblasts and smooth muscle cells were clustered based on their MMP expression to a resolution that generated 8 groups. Cells identified as fibroblast or smooth muscle are indicated at the top of the graph. **B)** Differentially regulated genes in the mesenchymal cell MMP clusters. The MMP clusters 0 to 7 are indicated at the bottom of the graph. Gene blocks associated with the different clusters are shown on the left. **C)** Fibroblast types based on gene enrichment. Genes associated with the different MMP clusters were analyzed using the ToppCell Atlas. The most significant association for each gene list is shown.

Since the CAFs and the SMCs in the CRC single-cell data expressed a similar set of MMPs, these two cell types were combined and resolved into 8 clusters (Fig 2A). The SMCs and CAFs equally populated many of the clusters, consistent with these two cell types expressing similar MMPs. However, clusters 3, 4 and 5 were mostly CAFs. These clusters were distinguished by high MMP1/3, high MMP11 and high MMP1 expression, respectively. To determine whether the MMP expression patterns were associated with different CAF types, genes enriched in the MMP-defined clusters were identified (Fig 2B) and analyzed for cell-type using the ToppCell Atlas database (Fig 2C). This analysis returned different cell categories for the different MMP-defined clusters, including “fibroblast-pericyte”, “mesenchymal fibroblast” and “myofibroblast” (Fig 2C). This analysis is consistent with different fibroblast types within CRC having unique MMP expression profiles, which likely impacts their role in regulating ECM dynamics in the tumor [9].

### MMP expression in myeloid cells

**Figure 3:**
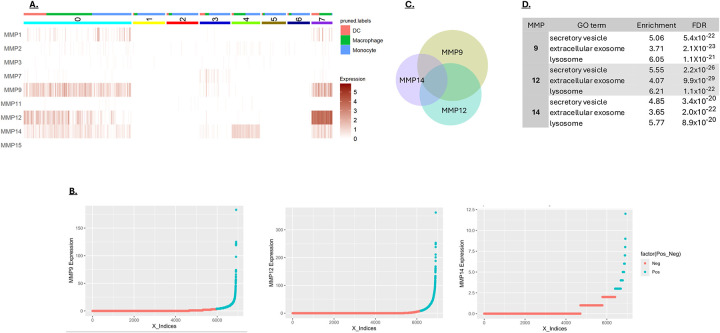
**A)** MMP-based clustering of myeloid cells from CRC. Monocytes, macrophages and dendritic cells (DC) from CRC were clustered based on their MMP expression to a resolution that generated 8 clusters. The cell type is indicated at the top of the graph. **B)** Distribution of MMPs 9, 12, and 14 expression levels in cancer-derived myeloid cells. Cells counted as positive are shown in turquoise, exhibiting expression above the median of expressing cells. **C)** Overlapping expression of MMP 9, 12 and 14 in cancer-derived myeloid cells. The size of the circles and the area of overlap are proportional to the number of MMP-expressing cells. **D)** Myeloid cells expressing MMPs 9, 12 or 14 show significant enrichment for genes associated with secretory vesicles, extracellular endosomes and lysosomes, based on the Gene Ontology (GO) Biological Processes database.

Cluster analysis of the myeloid-associated MMPs 9, 12 and 14 in the scRNA-seq data was performed focusing on three major myeloid cell types: monocytes, macrophages, and dendritic cells. The largest cluster (cluster 0) showed expression of all three myeloid-preferred MMPs in all three of the myeloid cell types (Fig 3A). Clusters 1, 2, 3, 5 and 6 showed relatively low MMP expression and were predominately populated with monocytes (Fig 3A). Two other clusters of interest are cluster 4, in which monocytes and macrophages express MMP14 almost exclusively, and cluster 7, in which macrophages and dendritic cells express high levels of MMP 12. To better understand the basis of MMP expression in the myeloid cells, high-expressing cells were resolved from low-expressing cells (Fig 3B). Significant overlap in expression of the three myeloid MMPs was observed, consistent with an underlying coregulation (Fig 3C). Genes associated with high MMP 9, 12 or 14 expression were analyzed for enrichment using the Gene Ontology database (GO). All three of the MMPs were associated with the expression of genes involved in secretory vesicle formation and the lysosome activity (Fig 3D). Based on this analysis, MMP expression in myeloid cells does not appear to be highly cell-type specific but is instead associated with a more robust expression of endomembrane system components, including secretory vesicles and lysosomes. Overall, this suggests that MMP expression in myeloid cells is associated with a more secretory phenotype.

### MMP expression in cancer cells

**Figure 4:**
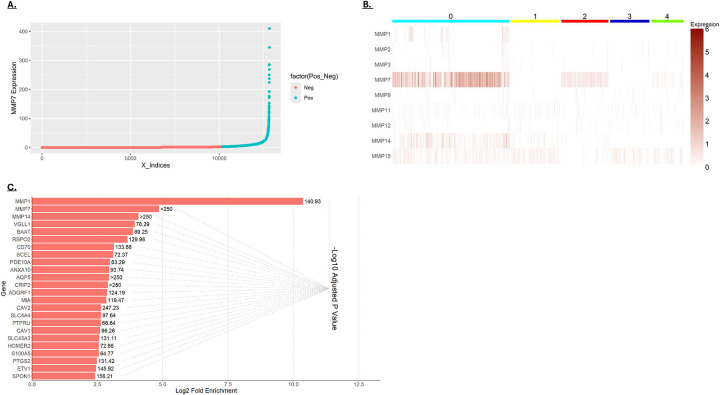
**A)** Distribution of MMP7 expression in individual cancer cells. The cells shown in turquoise were considered positive for MMP7 expression, expressing above the median of all MMP7-expressing cells. **B)** Cancer cells were clustered based on MMP expression at a resolution that generated 5 clusters. MMP7 expression was most robust in cluster 0. **C)** The most enriched genes in cluster 0 versus all other clusters (based on fold-enrichment). The graph shows the log_2_-fold enrichment and the −log_10_ of the adjusted *p*-value).

The scRNA-seq data showed that MMP expression is low in normal colonic epithelial cells but increases in colorectal cancer, with MMP7 showing high expression in a subset of cancer cells (Fig 1C)[10, 11]. As shown in Fig 4A, MMP7 expression in cancer cells varies by several orders of magnitude. To better understand the nature of MMP7-expressing cancer cells, cancer cells were clustered based on their MMP expression. Cancer cells expressing the highest MMP7 were found in cluster 0 (Fig 4B). To determine how MMP7 expression correlated with other cancer cell characteristics, genes enriched in cluster 0 were identified (Fig 4C). The most highly enriched genes in this cluster were MMP1, MMP7, and MMP14. This finding indicates that these cancer cells likely possess high ECM-degrading potential, possibly affecting their malignancy [10, 11]. Additionally, this cluster shows enriched expression of the Wnt-signaling enhancers RSPO2, SPON1, and PTGS2 [12–14], and the Wnt target genes MMP17, PTGS2/COX2 and ETV1 [15–17]. This observation is consistent with reports that MMP7 is itself a Wnt target gene and suggests that this subpopulation of cancer cells has elevated Wnt signaling [10, 18, 19].

### Spatial MMP expression in CRC

**Figure 5:**
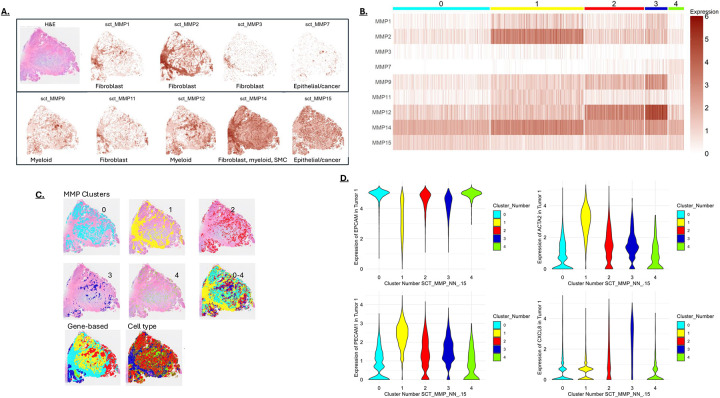
**A)** MMP expression mapped on a colon cancer section referred to as “tumor 1”. An H&E-stained section is shown in the upper-left panel. The primary cell types associated with the different MMPs (from Figure 1) are indicated at the bottom of each panel. **B)** MMP expression patterns in the pixels of tumor 1 were clustered at to generate 5 groups. **C)** MMP clusters from Figure 5B displayed on the tumor section, both individually and combined (0–4). Also shown are clusters based on the most differentially expressed genes (Gene-based) and Cell Type. MMP clusters resemble clusters generated from differentially expressed genes and cell type clustering. **D)** Violin plots showing selected marker genes in the MMP clusters. Epithelial/EPCAM-positive cells are a major component of clusters 0, 3 and 4, the ACTA2 activated fibroblast marker is highest in cluster 1, the angiogenesis marker PECAM1 is highest in cluster 1, and expression of the CXCL8 chemokine is highest in cluster 3.

To determine how different MMPs might interact within a CRC, we analyzed spatial transcriptomic data. Moderate resolution data with approximately 20 cells per pixel was used to reveal MMP expression overlap. Analysis of spatial transcriptomic data showed distinct regional expression patterns for individual MMPs (Fig 5A). To understand how MMPs expression overlaps within the lesion, MMP expression was clustered (Fig 5B) with these clusters “painted” on the tissue section (Fig 5C). The MMP expression clusters were driven in part by the cell types present in the pixel (Fig 5C). The MMP clusters also generated a patten similar to that made by the top differentially expressed genes (Fig 5C), a finding consistent with MMP expression helping to establish discreet subdomains. Marker gene analysis showed that most cancer cells were in MMP clusters 0, 3 and 4 (EPCAM-expressing cells), whereas most fibroblasts were in cluster 1 (ACTA2 cells) and most inflammatory cells were in cluster 3 (CXCL8-expressing cells)(Fig 5D). Cluster 1 also appears to be more angiogenic, based on PECAM1 expression (Fig 5D). Gene enrichment within the MMP clusters indicated that most cell division occurs in the cancer cell clusters 0 and 4, while fibroblastic cluster 1 is active in ECM remodeling and vascular development. Finally, cluster 3 shows enrichment in immune and inflammatory cell activity (Table 1), which formed discreet immune/inflammatory hubs in the lesion (Fig 5C).

As discussed above, cancer cells expressing MMP7 and other MMPs are potentially more prone to move through the ECM [20–23]. The cancer cell-specific MMP7 was split into multiple clusters (mostly clusters 3 and 4) with cluster 3 including high levels of the myeloid MMPs. These data show that a subpopulation of cancer cells with high MMP7 expression reside within a region that is also populated with MMP-expressing myeloid cells. This overlap of MMP expression may portend a higher ECM degrading potential and enhanced cancer cell movement through, and potentially out, of the primary lesion.

**Figure 6:**
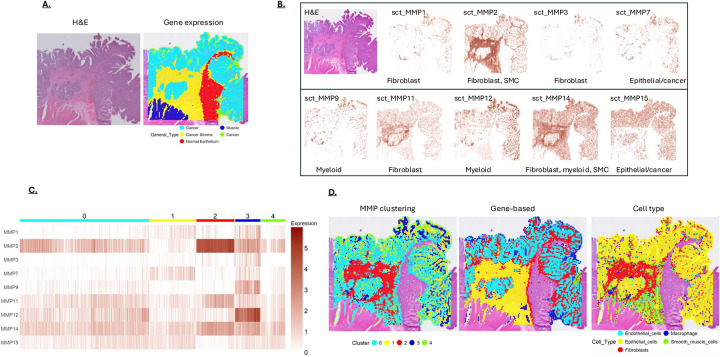
**A)** H&E staining shows cancerous tissue present in “tumor 2”. Non-cancerous regions were also revealed by gene expression analysis, which were removed prior to MMP cluster analysis for better comparison to tumor 1. **B)** MMP expression mapped on a section of “tumor 2”. An H&E-stained section is shown in the upper-left panel. The primary cell types associated with the different MMPs (from Figure 1) are indicated at the bottom of each panel. **C)** MMP expression in tumor 2 clustered at a resolution to generate 5 clusters. **D)** MMP clusters from Figure 6C displayed on the tumor section. Also shown are clusters based on the most differentially expressed genes (Gene-based) and Cell Type. Note the MMP clusters show a similar pattern to the gene-based and cell type clustering.

A second CRC was analyzed for spatial MMP expression (tumor 2). The tumor 2 tissue section H&E showed regions of smooth muscle and normal-appearing crypts, which were also revealed in the transcriptome analysis (Fig 6A). To be consistent with the tumor 1, these non-cancerous tissue regions were excluded from the analysis. Mapping of MMP expression on tumor 2 showed pattern similar to that found in tumor 1, with MMP expression dictated in part by the location of different cell types (Fig 6B). MMP clustering for tumor 2 is shown as a heat map in Fig 6C and “painted” on the tissue in Fig 6D. Cancer cells were found to predominate in clusters 0 and 1, with cluster 1 showing higher MMP7 expression and greater proliferative activity (Table 2), consistent with MMP7 marking more aggressive cancer cells [24–26]. As in tumor 1, dense stroma formed a distinct MMP2-high cluster (cluster 2), and the myeloid-expressed MMP9 and MMP12 grouped together in an immune/inflammatory cluster (cluster 3)(Table 2). Despite similarities with tumor 1, two differences were noted in tumor 2: 1) whereas the stromal region of tumor 1 showed high levels of MMP1 expression, relatively little MMP1 expression was found in the stromal region of tumor 2, and 2) tumor 2 showed a cluster with extensive MMP overlap, where fibroblast (MMP1, MMP3), myeloid (MMP9, MMP12), and cancer cell (MMP7) enzymes colocalized (cluster 3). The overlap of MMP expression in cluster 3 suggests an area with high ECM remodeling potential, which may facilitate ECM dynamics and cell movement. This may be particularly relevant since MMP7-expressing cancer cells are also found in this cluster and maybe more prone to move through the ECM.

### Variable MMP1 expression in CAFs

**Figure 7:**
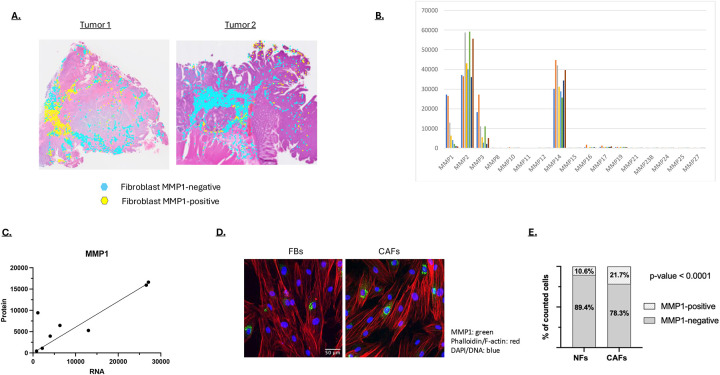
**A)** The fibroblast-dense regions in tumors 1 and 2 are shown in turquoise with the MMP1-expressing fibroblast regions shown in yellow. **B)** MMP expression in eight primary fibroblast cell lines generated from human colon cancers as determined by RNA-seq. **C)** MMP1 RNA expression level correlated with MMP1 protein expression. Secreted MMP1 was quantified by immunoblotting. **D)** MMP1 expression in fibroblasts isolated from normal colon (FBs) and cancers (Cancer associated fibroblasts, CAFs) visualized by immunofluorescent staining (green). F-actin/phalloidon staining is shown in red and DNA/DAPI staining shown in blue. **E)** Percent MMP1 expression in normal colonic fibroblasts (NF) and cancer associated fibroblasts (CAFs) based on immunofluorescent staining

One difference between tumors 1 and 2 was the MMP1 expression level in the stromal/fibroblast region. This is shown more clearly on the two tumor sections in Fig 7A, where fewer MMP1-positive fibroblast pixels are observed in tumor 2. To better understand MMP1 regulation in the fibroblasts, we quantified MMP RNA expression in eight CAF lines derived from eight CRC patients. The cultured CAFs expressed most of the MMPs observed in the single-cell RNA-seq analysis: MMP1, MMP2, MMP3, and MMP14 (Fig 7B; Fig 1C–D). Interestingly, MMP11 expression was detected in the scRNA-seq data but not in the cultured CAFs, suggesting that this fibroblast subtype is under-represented in the cultures (Fig 1C; Fig 7B). Among the CAF-expressed MMPs, MMP1 showed the greatest variability. MMP1 mRNA levels in the CAF lines corresponded closely to MMP1 protein levels, indicating that MMP1 regulation is primarily transcriptional (Fig 7C). Immunofluorescence staining for MMP1 protein revealed that only a subpopulation of CAFs expressed MMP1 (Fig 7D–E). For cells isolated from one patient, the proportion of MMP1-positive CAFs was higher than in fibroblasts cultured from adjacent normal tissue, consistent with the RNA-seq data (Fig 1).

**Figure 8:**
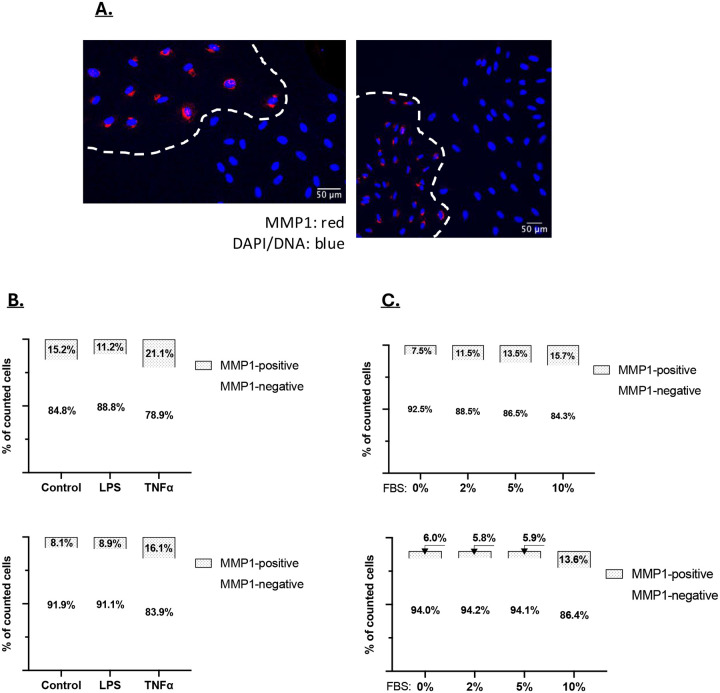
**A)** CAFs plated at low density to generate colonies were stained for MMP1 expression. MMP1 staining is shown red and DAPI/DNA staining is blue. Staining indicates that CAF colonies are either MMP1 positive or MMP1 negative. **B and C)** Two CAF lines were treated with LPS or TNF **(B)**, or with increasing concentrations of FBS **(C)**. The fraction of MMP1-positive cells was then determined by immunofluorescent staining.

To determine whether MMP1 expression constituted a stable CAF phenotype, several CAF lines were plated at low density to form discreet colonies. Immunofluorescent analysis showed that these colonies were either MMP1-positive or MMP1-negative. Fig 8A shows two examples where MMP1-positive and negative colonies were in close proximity. Given the size of the colonies, we estimate that the MMP1-phenoptype is stable through at least 5 cell divisions. The number of MMP1 staining cells varied between isolated CAF lines, ranging between 5 and 20% (Fig 8B). To determine whether the number of MMP1-expressing cells changes following stimulation, cultures were treated with TNF, LPS or increasing levels of serum, followed by MMP1 expression analysis. TNF and high serum concentrations were able to increase the fraction of MMP1-expressing cells to a degree, whereas LPS did not (Figs 8C and 8D). These data show that stimuli can increase the number of MMP1-expressing CAFs, but that the number of cells that can be converted is limited. Overall, these data indicate that MMP1-expression is a relatively stable fibroblast phenotype that persists upon cell division. These fibroblasts may constitute the “mesenchymal” fibroblast type identified in the scRNA-seq analysis (Fig 2C). These fibroblasts may proliferate within the cancer to generate the clusters of MMP1-expressing cells observed in the spatial transcriptomic data of tumor 1 (Figs 5A and 7A).

## Discussion

Here, we provide a comprehensive analysis of the cell-type-specific expression of MMPs in CRC. Distinct MMP expression patterns were observed across different cell types, and although individual cells can express MMPs outside their primary profiles, obvious preferences were observed. Within each major cell category, additional layers of specificity were uncovered, related to cell subtype, activation status, or a combination of both. Although many of our observations have been previously reported for individual cell types, our analysis serves to integrate this information while also providing an analysis of spatial expression patterns in CRC.

CAFs are high MMP expressors in CRC and can be resolved into multiple MMP-based clusters. Analysis of these clusters suggests that different fibroblast subtypes express distinct MMP combinations. This observation aligns with previous findings that CRCs contain multiple CAF types, including resident fibroblasts, fibroblasts derived from infiltrating mesenchymal stem cells, and cancer cells that have undergone an epithelial-to-mesenchymal transition [9, 27, 28]. Precisely how the MMP-based clusters identified here relate to the CAF subtypes described by others will require a more detailed analysis. In contrast to the fibroblasts, all myeloid cell types express the same set of MMPs: MMPs 9, 12 and 14, although monocytes tend to express lower levels than macrophages and dendritic cells. MMP expression levels in the myeloid cells correlates with the expression of genes related to secretory vesicle production, which is the route through which MMPs are secreted. In this regard, the MMP expression profile of myeloid cells appears to be related to a cell’s secretory activity rather than their type or subtype (aside from less-frequent expression in monocytes). Analysis of the spatial data shows that MMP-expressing myeloid cells form immune/inflammatory cell hubs within the CRC, which is likely to facilitate their cross signaling [29, 30].

The MMP most characteristically expressed in cancer cells is MMP7, a matrilysin with a broad spectrum of substrates. The expression level of MMP7 cancer cells ranges several orders of magnitude, with expression detected in approximately one-third of all cancer cells. Interestingly, a subset of cancer cells expressing high levels of MMP7 also express high levels of MMP1 and MMP14. These “triple MMP-positive” cancer cells might be more prone to passage through the ECM. Although it is not entirely clear how the expression of these MMPs is being regulated, MMP7 has previously been reported to be a target of the Wnt signaling pathway [31–33]. Concordant with this possibility, three Wnt signaling enhancers, RSPO2, SPON1 and PTGS2/COX2, are also expressed in the MMP7-high cluster, along with the Wnt-regulated genes MMP17, PTPRU, PTGS2/COX2 and ETV1 [12, 34–39]. The signals regulating this suite of genes may be a central determinant of metastatic potential.

Analysis of CRC spatial transcriptomic data showed MMP clusters consistent with the cell type expression derived from the scRNA-seq analysis. MMPs expressed primarily by cancer cells localized to regions of high cancer cell density, while fibroblast-derived MMPs were enriched in stromal areas. Myeloid cell-expressed MMPs clustered in regions characterized by immune and inflammatory gene expression. Computational clustering based on MMP expression yielded subregions within tumors that overlapped with regions defined by differential gene expression and cell type. This finding supports the idea that MMP activity contributes to the establishment and maintenance of tumor subdomains. Our findings show that fibroblast/stromal expressed MMPs are elevated in regions of active angiogenesis, suggesting that this combination or “cocktail” of MMPs may facilitate new vessel formation [40]. Likewise, the spatial enrichment of the myeloid MMPs may play a role in establishing immune and inflammatory cell hubs within the tumor [30].

Comparing the spatial MMP expression patterns of two tumors revealed similar clustering patterns, with some key differences. One difference of note was the degree of MMP overlap within the tumor. Since MMPs are active on different ECM substrates, the overlap of MMP expression within a tumor is likely relevant to lesion progression. Although only two tumors were spatially analyzed, different degrees of MMP overlap were observed. Analysis of additional tumors would be required to understand the relationship between spatial MMP expression overlap and the clinical characteristics of a CRC, but our data indicate that this analysis could be illuminating. Another notable difference observed was the level of MMP1-expression within the fibroblast-rich stromal regions. Our spatial analysis pointed to large differences in the number of MMP1-expressing CAFs in the two tumors analyzed. Analysis of CAFs cultured from patients likewise showed a broad range of MMP1 expression. Considering both the scRNA-seq data, and the clonal expression of MMP1 in cultured cancer fibroblasts, MMP1-expressing CAFs appear to be a cell type that integrates into, and proliferates within, the tumor. How the differences in fibroblastic/stromal MMP expression impacts tumor progression is not clear and will require the analysis of additional cancers with known clinical outcomes.

There are several caveats to our studies, the most obvious being that they are limited to RNA expression analysis. This limitation is particularly important for MMPs, as they can be regulated at multiple levels, including secretion, zymogen activation, and inhibition by TIMPs. Nevertheless, our findings have implications for understanding tumor progression and for potential therapeutic strategies. Although MMP inhibitors showed promise in preclinical studies, most have failed in clinical trials due to excessive toxicity and limited efficacy [41]. Broad-spectrum inhibitors such as batimastat and marimastat caused significant musculoskeletal toxicity, whereas more selective agents like DX-2400 (targeting MMP-14) raised concerns because MMP14 is expressed in both tumor and normal tissues [42]. Identifying which MMPs are selectively enriched in tumors may help refine therapeutic targeting. Our analysis indicates that MMPs 1, 3, 7, and 11 are cancer-enriched, whereas MMP2, MMP14, and MMP15 are not. Understanding the spatial deployment of MMPs may also aid in the development of selective inhibitors that minimize off-target effects. For instance, targeting MMPs expressed in angiogenic regions may synergize with VEGF inhibitors such as bevacizumab to more effectively suppress neovascularization. Beyond inhibition, MMP expression has also been proposed as a means of therapeutic targeting, either through localized drug release or antibody-based strategies. MMP2-cleavable nanoparticles have been shown to successfully deliver drugs such as SN38 and doxorubicin in CRC models [43–46]. Our findings suggest that MMP7 could be leveraged for targeted delivery directly adjacent to aggressive cancer cells. Our analysis also highlights which MMPs might not be helpful to target. For example, MMP12, which is expressed primarily in cancer-associated myeloid cells, has been implicated in tumor suppression [47]. This MMP may be important for dendritic cell function. Overall, our findings demonstrate that there is still much to learn about the role and deployment of MMP enzymes in CRC. This information could facilitate improved prognosis and potentially novel therapeutic targeting approaches.

## Methods

### Data acquisition and processing

Bulk RNA-seq data for identifying MMPs were obtained from the TCGACOAD STAR cohort. Single-cell RNA-seq data were retrieved from GEO (GSE132465), and spatial transcriptomic datasets were obtained from 10X Genomics (CytAssist_11mm_FFPE_Human_Colorectal_Cancer, Tumor 1; Visium_HD_Human_Colon_Cancer, Tumor 2). All analyses were performed in R. Bulk RNA-seq counts from the TCGACOAD cohort were normalized using DESeq2, and differential MMP expression between cancer and normal tissues was visualized with ggplot2. Highly expressed MMPs were further examined in single-cell and spatial datasets. Single-cell data were processed as Seurat objects using SCTransform, with celltype annotation via SingleR using the HumanPrimaryCellAtlas dataset (restricted to CRC-relevant types) as reference. Data were subset into epithelial, myeloid, and fibroblast/smooth-muscle compartments. Clustering followed guidelines established by Satija Lab’s Guided Clustering Tutorial, using Seurat’s RunPCA, FindNeighbors, and FindClusters functions. Clustering was carried out based both on variable genes and selected MMP genes. Nearest-neighbor and shared-nearest-neighbor approaches across multiple resolutions provided complementary cluster perspectives.

### Spatial data processing

The spatial transcriptomic data from the two colon cancers were captured at different resolutions. To make the datasets comparable in pixel number and reads per pixel, the 8 μm × 8 μm bins of tumor 2 were aggregated into 56 μm × 56 μm bins. Both tumors were processed as Seurat objects and clustered using variable gene and MMP-based approaches. Pixel level cell-type assignments used SingleR with the same reference as single-cell data. Fibroblast and smooth-muscle–associated pixels were further subset for focused analyses.

### Visualization and downstream analyses

Dot plots, heatmaps, violin plots, and bar charts were generated with ggplot2. Cells were classified as positive or negative for a gene based on the median expression of all expressing cells. SpatialFeaturePlot and SpatialDimPlot were used to visualize spatial MMP expression and clustering. One additional gene expression heatmap was created with ComplexHeatmap. Cluster-defining genes were identified using Seurat’s FindAllMarkers function. The top 500 positive markers from each cluster were analyzed with PANTHER Gene Ontology for Biological Process. Enrichment, probability, and representative processes were summarized in gt-generated tables.

### Fibroblast isolation and culture

Colorectal tissue specimens were obtained from UConn Health/Waterbury Hospital. All samples and associated clinical data were collected from consented patients undergoing colon resection and were de-identified in accordance with institutional protocols and established ethical guidelines. Tissues were washed with 1x Hanks’ Balanced Salt Solution and incubated with 25 mM ethylenediaminetetraacetic acid (EDTA) for 1 hour at 4o C. Epithelial cell layer was stripped off by multiple rounds of vortexing. Remaining tissues were incubated with collagenase solution (1 mg/ml collagenase, 1 mg/ml dispase and 10ug/ml DNase I) for 90 min at 37o C. Digested tissues were filtered (70 mm), centrifuged at 300g for 5 min and washed with Dulbecco’s Modified Eagle Medium (DMEM) media supplemented with 10% fetal bovine serum (FBS), 2 mM L-glutamine and 1x Penn/Strep solution (Gibco). Resuspended cells were cultured in complete DMEM, and frozen in aliquots with complete medium plus 10% dimethyl sulfoxide (DMSO). For growth and analysis, frozen cell aliquots were thawed and maintained in Lonza fibroblast growth medium (FGM-2 Fibroblast Growth Medium-2 BulletKit) at 37°C in a humidified atmosphere with 5% CO2. Cells were subcultured for up to seven passages and monitored for the changes in morphology.

### Fibroblast treatments and analyses

To assess MMP1 expression in response to stimuli, cells were grown on coverslips, serum-starved overnight, and then treated with 10 ng/mL TNFα (Thermo Fisher Scientific), 1 μg/mL LPS-EB (InvivoGen), or graded serum levels for 20 h. To assess differences in MMP1 expression between fibroblast colonies, cells were seeded on coverslips in 24-well plates at a density of 120 cells per well with the addition of conditioned medium collected from the cells’ previous plate (25% v/v). Cells were incubated for 9 days, with medium replenished as needed. For the RNA-seq analysis, RNA was isolated using RNeasy extraction kit (Qiagen), according to manufacturer’s instructions. RNA quality assessment was performed at the Center for Genome Innovation (CGI) core facility at the University of Connecticut. Library preparation and bulk RNA sequencing were performed by Azenta Life Sciences on an Illumina NovaSeq 6000 platform, generating 150 bp paired-end reads with a target depth of ~30x. Data were normalized and analyzed using DESeq2 (R/Bioconductor) to quantify gene expression changes. For immunofluorescence staining experiments, cells were fixed in 3% paraformaldehyde, permeabilized with 0.3% Triton X-100 in the Dulbecco’s phosphate-buffered saline (DPBS), and blocked in 5% Goat serum (Jackson ImmunoResearch) in DPBS. Cells were then stained for MMP1 expression using the GeneTex MMP1 antibody GTX100534 and appropriate secondary antibodies (Thermo Fisher Scientific, Jackson ImmunoResearch). Nuclei were visualized using DNA dye DAPI (Thermo Fisher Scientific). Fluorescent images were acquired with a Nikon A1R laser scanning confocal microscope (Nikon Instruments, Japan) and analyzed using Fiji/ImageJ software. Two-sided Fisher’s exact test (GraphPad Prism) was used to compare MMP1–positive cells between normal fibroblasts and CAFs; p < 0.05 was considered significant. Data were presented as percentages. Secreted MMP1 protein levels were quantified via Western blot using Mini-PROTEAN TGX precast gels (Bio-Rad), GeneTex MMP1 primary antibody, and IRDye^®^ 680RD secondary antibody. Blots were visualized on a LiCor Odyssey^®^ CLx Imaging System.

## Figures and Tables

**Table 1:** 

Tumor One *Go-Identified* Cluster Upregulated Processes *Using SCT_MMP_NN_.75 Clustering*			
Cluster Number	Processes	Fold Enrichment	Raw P Value
Cluster 0			
0	Chromosome Organization	5.62	1.93E-27
0	Cell Division	4.80	2.31E-25
0	Organelle Organization	2.01	9.18E-19
Cluster 1			
1	Cell Adhesion	4.74	7.95E-46
1	Vasculature Development	6.08	3.38E-42
1	Extracellular Matrix Organization	8.19	1.03E-36
Cluster 2			
2	Aerobic Respiration	5.78	1.22E-11
2	Response to External Biotic Stimulus	2.15	1.76E-10
2	Positive Regulation of Cell Migration	3.00	4,43E-10
Cluster 3			
3	Regulation of Immune System Process	3.69	1.97E-48
3	Inflammatory Response	5.38	9.43E-36
3	Regulation of Cell Motility	3.57	9.12E-27
Cluster 4			
4	Cell Division	4.96	1.41E-26
4	Nuclear Chromosome Segregation	6.72	2.97E-21
4	DNA Replication	5.69	1.03E-13

**Table 2:** 

Tumor 2 0-1-4 Subset *GO-Identified* Cluster Upregulated Processes *Using SCT.MMP_SNN_.05 Clustering*			
Cluster Number	Processes	Fold Enrichment	Raw P Value
Cluster 0			
0	Cell Migration	2.24	5.98E-8
0	Tube Development	2.23	9.58E-8
0	Circulatory System Development	2.14	4.70E-7
Cluster 1			
1	Nucleobase-Containing Compound Metabolic Process	1.80	2.90E-11
1	Regulation of Cell Population Proliferation	2.00	1.10E-9
1	Cell-Cell Junction Organization	4.75	1.55E-9
Cluster 2			
2	Extracellular Matrix Organization	9.87	4.15E-50
2	Circulatory System Development	4.61	1.42E-42
2	Collagen Fibril Organization	17.31	4.88E-48
Cluster 3			
3	Immune Response	3.71	1.30E-51
3	Leukocyte Activation	5.99	9.78E-47
3	Inflammatory Response	6.02	7.01E-44
Cluster 4			
4	Organic Acid Catabolic Process	5.29	9.48E-14
4	Fatty Acid Metabolic Process	3.78	2.61E-10
4	DNA Replication	4.40	6.66E-9
